# Editorial: Advances in brain dynamics in the healthy and psychiatric disorders

**DOI:** 10.3389/fpsyt.2023.1284670

**Published:** 2023-09-14

**Authors:** David Papo, Maide Bucolo, Stavros I. Dimitriadis, Julie A. Onton, Athineos Philippu, David Shannahoff-Khalsa

**Affiliations:** ^1^Center for Translational Neurophysiology of Speech and Communication, Fondazione Istituto Italiano di Tecnologia, Ferrara, Italy; ^2^Department of Neuroscience and Rehabilitation, Section of Physiology, University of Ferrara, Ferrara, Italy; ^3^Department of Electrical, Electronic and Informatics, University of Catania, Catania, Italy; ^4^Department of Clinical Psychology and Psychobiology, Faculty of Psychology, University of Barcelona, Barcelona, Spain; ^5^Institute of Neural Computation, University of California, San Diego, La Jolla, CA, United States; ^6^Department of Pharmacology and Toxicology, University of Innsbruck, Innsbruck, Austria; ^7^BioCircuits Institute, University of California San Diego, La Jolla, CA, United States; ^8^Center for Integrative Medicine, University of California San Diego, La Jolla, CA, United States; ^9^The Khalsa Foundation for Medical Science, Del Mar, CA, United States

**Keywords:** brain dynamics, brain thermodynamics, time-reversal symmetry, fluctuation-dissippation relations, brain fluctuations, dynamical disease

Psychiatry aims at diagnosing and treating psychological disorders and other mental health conditions affecting how subjects behave, think, or feel. Psychiatrists resort to a variety of diagnostic techniques, ranging from standard semiotics to physiological testing, and imaging or stimulation techniques, and treatment strategies may be behavioral, pharmacological, or instrumental. However, knowledge of how neural activity translates into behavior is often insufficient to define precise nosological categories and to interact with the brain in a language it can understand. Improvements in both modeling and treatment require a better understanding of the underlying neural processes and finding new meaningful variables to characterize both healthy brain activity and its pathology.

The brain is, in general, thought of as a spatially-extended dynamical system subject to some control parameter. This framework allows not only describing and to some extent predicting healthy (1) and pathological (2–4) brain activity and treatment outcomes (5), but also maintaining activity within or steering it toward desirable dynamical regimes (6). The challenge is specifying the dynamics and identifying its control parameters and the appropriate strategies allowing to effectively act on the system (6).

Dynamical systems can be studied in three main ways: perturbing the system, observing its unperturbed dynamics, and characterizing its symmetries. *Prima facie*, the most sensible strategy to quantify the neural correlates of psychiatric disorders would seem to require observing task-related brain activity and behavior. However, finding appropriate tasks, capable of testing individuals with tasks they can perform (7) is not always easy, particularly when testing dynamically complex brain functions (8). Moreover, this strategy is necessarily associated with lengthy (possibly anxiogenic) testing sessions, whose neural correlates may be hard to gauge, and whose time and economic costs can also be sizeable.

An alternative strategy involves extracting meaningful properties from spontaneous brain activity. Spontaneous activity can be thought of as a data bank of cortical fluctuation patterns (9) with complex spatio-temporal structure (10), displaying similar non-trivial properties across a wide range of scales (11, 12). These properties are replicated in behavioral fluctuations (13), suggesting that behavior is in essence a coarse-grained version of neural fluctuations. Importantly, these properties are altered in various brain pathologies (14, 15) and can be modulated by pharmacological manipulations (16). These fluctuations are intrinsically multiscale with complex relations among scales (17, 18), so that characterizing the temporal scales of cognitive processes is in general non-trivial (19, 20).

These fluctuations can be thought of as the statistical and dynamical signatures of underlying non-linear dynamical processes in terms of which the system can be described, and the behavioral or neural variables capable of modulating them as control parameters for the dynamics (10).

Dynamics and thermodynamics and, more specifically, non-linearity and non-equilibrium properties constitute two sides of the same coin. This can be appreciated by considering symmetries and their break down. For instance, the breakdown of time-reversal symmetry, a measure of the extent to which it is possible to discern a preferred time direction of a stationary stochastic process (21), which is associated with the presence of strong non-linearities (22), constitutes the hallmark of systems operating away from equilibrium (23). These systems use part of their free energy budget to perform work or store energy in alternative forms, dissipating the rest as heat in the environment. The second law of thermodynamics prescribes that this transformation should be associated with an irreversible increase in entropy of the environment. The higher the price in entropy lost to dissipation, the more conspicuous the irreversibility. Thus, time-reversal symmetry can be used not only as an indicator of whether a system is at equilibrium or not (24, 25), but also as a quantifier of its distance from such a condition (26). Importantly, irreversibility can be quantified from experimental data (27). Not surprisingly, the marked irreversibility of healthy spontaneous brain activity (22, 28, 29) shows specific alterations in various conditions (28–31), including Alzheimer's disease (28), ADHD (29), bipolar disorder (29), and schizophrenia (28, 29).

Dissipation is also proportional to the violation of *fluctuation-dissipation relations* (FDRs) (32), expressing fundamental symmetries of equilibrium systems (33). In such systems, the autocorrelation of some observable's fluctuations in the unperturbed system is related through temperature to the response to small external perturbations. Brain fluctuations are profoundly different from the Gaussian ones with exponentially vanishing memory of equilibrium systems (34–37), and this relationship must be expressed differently, e.g., close to equilibrium, in terms of an effective temperature (38).

Mirroring dynamics' multiscaleness, irreversibility and FDRs' violations may manifest differently at different spatial and temporal scales (39–41). Accordingly, brain activity and its pathology can be described in terms of the characteristic scales of such properties (10, 28, 42).

Insofar as the brain ultimately manipulates information, one may want to quantify brain activity in terms of information processing, erasure, and transfer. A deep relation exists between information and thermodynamics of a physical system (43, 44). In particular, the Landauer principle states that information erasure is a dissipative process (45), Likewise, *effective information use* is related to *thermodynamic efficiency*. This is because neural systems compute implicit models of the environment through their dynamics. However, a fraction of retained information does not improve the system's predictive power and is equivalent to *thermodynamic inefficiency* (46) (see Figure 1).

**Figure 1:**
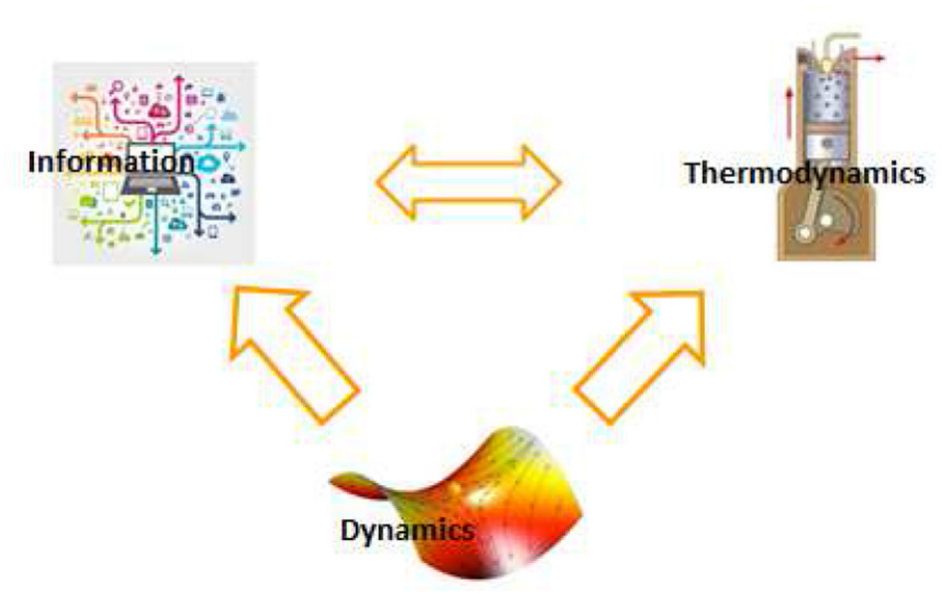
Brain activity in healthy and pathological states are traditionally described by extracting functionally relevant features of its dynamics. Complementary descriptions can be found by using the relationships between dynamics, thermodynamics, and information. Ultimately, brain activity and its pathologies can be characterized in terms of physically meaningful dynamical, thermodynamical and information theoretic variables (10).

This Research Topic presents five contributions dealing with various aspects of psychiatric pathology, particularly of obsessive-compulsive disorder (OCD), but also unipolar depression, ranging from general characterization to symptom provocation, to treatment evaluation, and using various techniques (NIRS, MEG, TMS, but also behavioral techniques). Liu et al. found an association between OCD and brain aging acceleration. Bernardi et al. highlighted differences in the temporal scales of MEG activity's irreversibility in OCD with respect to healthy controls. Maia et al. propose a tutorial for TMS-guided symptom provocation in OCD. Stephenson et al. show the feasibility of an electronically delivered cognitive behavioral therapy program associated with functional neuroimaging evaluation. Finally, Yang et al. used NIRS to highlight topographically specific activations in first-episode vs. recurrent depression patients.

## Author contributions

DP: Conceptualization, Writing—original draft. MB: Writing—review and editing. SD: Writing—review and editing. JO: Writing—review and editing. AP: Writing—review and editing. DS-K: Writing—review and editing.
